# PPARG activation promotes the proliferation of colorectal cancer cell lines and enhances the antiproliferative effect of 5-fluorouracil

**DOI:** 10.1186/s12885-024-11985-5

**Published:** 2024-02-20

**Authors:** Leah Schöckel, Christine Woischke, Sai Agash Surendran, Marlies Michl, Tobias Schiergens, Andreas Hölscher, Florian Glass, Peter Kreissl, Frederick Klauschen, Michael Günther, Steffen Ormanns, Jens Neumann

**Affiliations:** 1 Institute of Pathology, Ludwig-Maximilians-University (LMU) München, Munich, Germany; 2 Department of Medicine III, University Hospital, LMU Munich, Munich, Germany; 3 Department of General, Visceral and Transplantation Surgery, University Hospital, LMU Munich, Munich, Germany; 4 Maria-Theresia-Klinik München, Munich, Germany; 5 District Hospital Ebersberg, Ebersberg, Germany; 6 German Cancer Consortium (DKTK), partner site Munich, a partnership between DKFZ and LMU Munich Germany, Munich, Germany; 7 Innpath Institute for Pathology GmbH, Tirol Kliniken, Innsbruck, Austria

**Keywords:** Colorectal cancer; metastasis, Diabetes mellitus, PPARG, 5-fluorouracil

## Abstract

**Background:**

Peroxisome proliferator-activated receptor gamma (PPARG) is a member of the nuclear receptor family. It is involved in the regulation of adipogenesis, lipid metabolism, insulin sensitivity, vascular homeostasis and inflammation. In addition, PPARG agonists, known as thiazolidinediones, are well established in the treatment of type 2 diabetes mellitus. PPARGs role in cancer is a matter of debate, as pro- and anti-tumour properties have been described in various tumour entities. Currently, the specific role of PPARG in patients with colorectal cancer (CRC) is not fully understood.

**Material and methods:**

The prognostic impact of PPARG expression was investigated by immunohistochemistry in a case-control study using a matched pair selection of CRC tumours (*n* = 246) with either distant metastases to the liver (*n* = 82), lung (*n *= 82) or without distant metastases (*n* = 82). Its effect on proliferation as well as the sensitivity to the chemotherapeutic drug 5-fluorouracil (5-FU) was examined after activation, inhibition, and transient gene knockdown of PPARG in the CRC cell lines SW403 and HT29.

**Results:**

High PPARG expression was significantly associated with pulmonary metastasis (*p* = 0.019). Patients without distant metastases had a significantly longer overall survival with low PPARG expression in their tumours compared to patients with high PPARG expression (*p* = 0.045). In the pulmonary metastasis cohort instead, a trend towards longer survival was observed for patients with high PPARG expression in their tumour (*p* = 0.059). Activation of PPARG by pioglitazone and rosiglitazone resulted in a significant dose-dependent increase in proliferation of CRC cell lines. Inhibition of PPARG by its specific inhibitor GW9662 and siRNA-mediated knockdown of PPARG significantly decreased proliferation. Activating PPARG significantly increased the CRC cell lines sensitivity to 5-FU while its inhibition decreased it.

**Conclusion:**

The prognostic effect of PPARG expression depends on the metastasis localization in advanced CRC patients. Activation of PPARG increased malignancy associated traits such as proliferation in CRC cell lines but also increases sensitivity towards the chemotherapeutic agent 5-FU. Based on this finding, a combination therapy of PPARG agonists and 5-FU-based chemotherapy constitutes a promising strategy which should be further investigated.

**Supplementary Information:**

The online version contains supplementary material available at 10.1186/s12885-024-11985-5.

## Introduction

Colorectal cancer (CRC) is one of the most common health problems in western industrialised countries and a major cause of cancer-related deaths [1, 2]. The heterogeneity of CRC with variable response to therapy makes easy-to-survey prognostic parameters desirable [3]. Peroxisome proliferator-activated receptors (PPAR) are members of the nuclear receptor superfamily. Three PPAR isotypes, each with a tissue-specific expression pattern, have been identified to date: PPARα, PPARβ/δ, and PPARG [4, 5]. Especially PPARG represents a promising target for patients with CRC [6]. PPARG forms heterodimers with retinoid X receptors (RXR) and recognizes specific sequence motifs, called peroxisome proliferator response elements (PPRE), in the regulatory regions of target genes [7, 8]. PPARG is involved in the regulation of adipogenesis, lipid metabolism, insulin sensitivity, vascular homeostasis and inflammation [5, 9]. Furthermore, the impact of PPARG on carcinogenesis is widely discussed in the literature. PPARG is highly expressed in 70% of sporadic CRC and expressed weakly in approximately 30% [10]. For PPARG ligands such as rosiglitazone and troglitazone an effect on cell cycle arrest, differentiation, proliferation, and migration of tumour cells could be observed [11, 12]. PPARG ligands reduce primary tumour growth and metastasis through inhibition of angiogenesis [13]. In the literature, there is also evidence that mutations in the PPARG gene are related to the development of neoplasia [14, 15]. In addition, PPARG agonists, known as thiazolidinediones, are well established in DM type 2 therapy and have relatively few side effects [16, 17]. With these background insights, the characterization of the biological function of PPARG in CRC seems promising. In the future, individualized therapy regimens besides the classical chemotherapeutic agents, which may have significant side effects, will become more important in the treatment of CRC. Currently, the specific role of PPARG in patients with CRC is incompletely understood. Therefore, we designed a case-control study of CRC patients to investigate the potential role of PPARG in this clinical setting. Additionally, we experimentally examined its effect on cell viability in colorectal cell lines.

## Material and methods

### Tissue collection

Two-hundred-and-fourty-six formalin fixed-paraffin embedded (FFPE) tissue samples from the archives of the Institute of Pathology, Faculty of Medicine, Ludwig-Maximilians-Universität (LMU) Munich were analysed from patients with CRC diagnosed between 1998 and 2017. For statistical reasons the study was designed as a case-control study with three different cohorts of 82 cases each, depending on the metastatic status of the patients. The first arm consisted of patients without distant metastases at the time of diagnosis and with a relapse-free survival of at least 5 years after primary surgical resection (termed M0). The second arm included patients with histologically or radiologically confirmed synchronous hepatic metastases (termed HEP). The third arm consisted of patients with histologically or radiologically confirmed synchronous pulmonary metastases (termed PUL). The cases in the three groups were matched in pairs according to tumour localization, tumour grade and T-category according to UICC. The appropriate clinicopathological data sets were acquired from the Munich Cancer Registry (MCR, Tumorzentrum München). The study was approved by the local ethics committee of the Medical Faculty of LMU Munich (project number: 20–104).

### Immunohistochemistry

To detect PPARG expression in FFPE tissue, a polyclonal rabbit anti-human PPARG specific antibody (LSBio, Seattle,USA) was used at a dilution of 1:100 after heat mediated epitope retrieval (target retrieval solution, Dako North Amerika Inc., Carpinteria, USA). Signals were detected using ImmPress Horse Anti-Rabbit IgG Kit (Vector Laboratories Inc., Burlingame, USA) and hematoxylin was employed for counterstaining (Vector Laboratories Inc., Burlingame, USA). Appropriate negative control tissue (normal exocrine pancreas) and positive control tissue (normal colonic mucosa) was included in each staining run.

### Scoring of immunohistochemistry

The evaluation of the immunohistochemical staining of PPARG was performed by two independent observers (JN, LS). The categorisation into high and low expression was done using the H-score [18]. Each tumour region was attributed an intensity value from 0 to 3 (where 0 represents no staining, 1 represents incomplete staining, 2 represents complete staining, and 3 represents complete staining with ascending intensity), and the proportion of tumour staining was recorded for that intensity in 5% increments from 0 to 100. The final score (possible range, 0–300) was calculated from the sum of the products of the intensity value and proportion of tumour staining. Cases showing an expression equal to or higher than the median (median = 140) were assigned to the high expression group, cases with scores lower than the median to the low expression group.

### Cell lines and reagents

Human CRC cell lines SW403 and HT29 were purchased from the German Collection of Microorganisms and Cell Culture (DSMZ, Braunschweig, Germany). Cell lines were maintained in Dulbecco’s Modified Eagle’s Medium DMEM (SigmaAldrich, Munich, Germany) supplemented with 10% fetal bovine serum (SigmaAldrich) and 1% penicillin/streptomycin (SigmaAldrich) and cultured with 5% CO_2_ at 37 °C. Cell lines were screened for mycoplasma contamination using a PCR based assay before each round of experiments. After thawing, cell lines were used until passage number 15 and then discarded. The PPARG agonists rosiglitazone and pioglitazone were purchased from Targetmol (Wellesley Hills, USA). The PPARG inhibitor GW9662 was acquired from SigmaAldrich. The different substances were diluted in DMSO (CarlRoth, Karlsruhe, Germany). 5-Fluorouracil (5-FU) was obtained from the pharmacy of the LMU hospital (Munich, Germany).

### siRNA-mediated gene knockdown

The transient suppression of the PPARG expression was carried out based on the principle of RNA interference using siPools [19], a mixture of up to 30 different small interfering RNA’s (siTools, Planegg, Germany), according to the manufacturer’s instructions. Briefly, siPools were diluted in OptiMEM (ThermoFisher Scientific, Schwerte, Germany), mixed with Lipofectamine RNAiMax (ThermoFisher Scientific) and subsequently added to the cell culture at a final concentration of the siPool of 2 nM. Twenty-four hours after the transfection the medium was changed and the cells were used for further experiments.

### Cell viability assays

A resazurin assay (SigmaAldrich) was used to measure metabolic activity as a well-established surrogate marker for proliferation in CRC cell lines. For all cell viability assays 96-well plates were used with a final volume of 100 μl. Due to different proliferation rates, 5000 cells per well were seeded for the SW403 cell line and 2000 cells per well for HT29 at the start of the experiment. The incubation period for all treatments (pioglitazone, rosiglitazone, GW9662, transient knockdown, control) was 72 hours. The assay was evaluated using a microtitre plate photometer (ThermoFisher Scientific) 4 hours after the addition of 10 μl of diluted resazurin reagent. The experiments involving PPARG inhibitors and activators were carried out in 12 replicates per experimental condition. The experiments involving chemotherapeutic agents were carried out in triplicates per condition. All experiments were repeated at three different timepoints, resulting in *n* = 36 or *n* = 9 samples per condition respectively.

### RNA extraction and cDNA synthesis

RNA was extracted from the cell lines using the NucleoSpin RNA Kit (Macherey-Nagel GmbH & Co. KG, Düren, Germany) according to the manufacturer’s instructions. RNA concentration was determined using a Nanodrop ND-1000 spectrophotometer (ThermoFisher Scientific). cDNA synthesis was performed using the RevertAid H minus First Strand cDNA Synthesis Kit (ThermoFisher Scientific) following the manufacturer’s instructions employing 1 μg of RNA.

### Quantitative real-time PCR

PPARG cDNA copies were quantified by quantitative PCR using a LightCycler 480 instrument (Roche, Penzberg, Germany) and normalised to the reference gene GAPDH. Samples were prepared for rt-PCR using the SYBR-Select Master Mix (ThermoFisher Scientific) according to the manufacturer’s instructions and transferred to a 96-well plate. The following thermal cycling protocol was used: denaturation 1 second at 95 °C; annealing 20 seconds at 60 °C; extension 1 second at 72 °C (40 cycles). Melting curves were evaluated for each experiment to confirm the generation of specific PCR products (10 seconds at 98 °C; 60 seconds at 60 °C, 5 minutes at 98 °C). All measurements were performed in biological triplicates and a final amount of 15 ng of cDNA was used for each sample. The ∆∆ CP method was used to calculate the final differences in PPARG expression (supplemental Fig. 1).

### Immunoblotting

The cultivated cells were washed in cold phosphate-buffered saline (PBS), scraped, and then lysed in triple lysis buffer [20] (50 mM Tris-HCl pH 8.0, 150 mM NaCl, 0.02% NaN3, 0.5% Na-Desoxycholat, 0.1% SDS, 1% Nonidet P-40, 10x Phosstop Phosphatase inhibitor cocktail, 7x Complete Protease inhibitor cocktail (both Roche)). After sonification for 5 seconds at an amplitude of 75%, the cell debris was removed by centrifugation and the protein concentration was estimated using the DC Protein Assay System (Biorad, Munich, Germany) according to the manufacturer’s instructions. Proteins were separated by 10% SDS-polyacrylamide gel electrophoresis and blotted on PVDF membranes (Millipore, Schwalbach, Germany). The membrane was blocked for 1 hour at room temperature in TBS/T buffer containing 5% non-fat dry milk (NFDM) (SERVA, Heidelberg, Germany), washed three times in TBS/T and incubated with the primary antibody dilutions overnight at 4 °C under constant agitation. The antibodies and their employed concentrations were as follows: PPARG (clone C26H12, Cell Signaling, Danvers, MA, USA) 1:1000 in TBS/T / 5% BSA; CK20 (clone Ks20.8, Medac Diagnostika; Wedel, Germany) 1:1000 TBS/T 5% NFDM, β-Actin (clone AC-15, SigmaAldrich) 1:30.000 in TBS/T 5% NFDM. After washing and incubation with horseradish peroxidase (HRP)-labelled secondary antibodies, the membranes were incubated with ImmobilonP Chemiluminescent Substrate (Millipore, Schwalbach, Germany) and chemiluminescence was detected using a digital imaging system (Li-COR Odyssey Fc, Lincon, NE, USA). The ratio of band density was calculated using ImageJ (supplemental Table 2).

### Statistics and data analysis

The significance of the correlations of the immunohistochemical analyses were tested with the x^2^-test. The paired t-test and Wilcoxon-Rank test were used for the statistical evaluation of the cell viability assays (*p*-values in brackets in the continuous text refer to the paired t-test). A PPARG-associated 5-FU resistance score and the correlation of PPARG expression with the expression of 5-FU-resistance associated genes was calculated in the R statistical environment as described previously [21]. Normalized RNAseq expression data was downloaded and heatmaps were generated as described before [22]. For all tests, a two-tailed a-error of less than 5% (*p* < 0.05) was regarded as statistically significant. Statistical significance is indicated as **** *p* < 0.0001, *** *p* < 0.001, ** *p* < 0.01, * *p* < 0.05, ns non-significant. The statistical analyses were performed using SPSS Version 26 (IBM, New York, USA).

## Results

### Correlation of PPARG with clinicopathological parameters

**Fig. 1:**
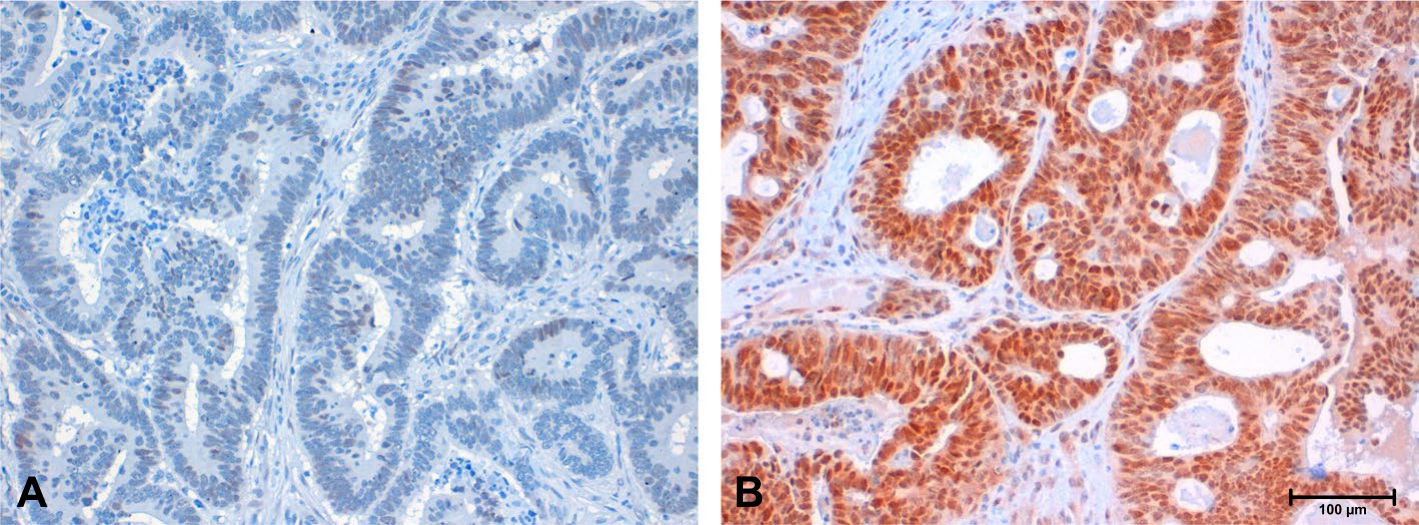
Immunohistochemical staining of PPARG in CRC (200-fold magnification). Tumour cells with low (**A**) or high (**B**) expression of PPARG based on the H-Score (scale bar indicates 100 μm)

To examine PPARG in patients with CRC, its expression was determined by immunohistochemistry (Fig. 1) in 246 FFPE tissue sections and subsequently correlated with the patients´ clinicopathological parameters. To quantify the expression of PPARG in this case-control study, the H-score was applied (Table 1). The PPARG expression level correlated significantly with the metastatic status of the patients (*p* = 0.009). CRC with pulmonary metastases showed the strongest PPARG expression with a mean of 156.9 and a median of 165, followed by the hepatic group with a mean of 128.1 and a median of 132.5. Patients without distant metastases had the lowest PPARG expression with a mean of 123.7 and a median of 122.5. For correlation with clinicopathological parameters, the expression of PPARG was categorised into low and high expression based on the median (Table 2). Expression of PPARG correlated significantly with gender (*p* = 0.005). In comparison to the M0 cohort, patients with isolated lung metastasis (PUL) showed significantly higher PPARG expression in their tumours (*p* = 0.019). In the analysis of the entire study population, PPARG showed a statistically non-significant trend towards a correlation with the status of distant metastasis (*p* = 0.053). No statistically significant differences in PPARG expression between the M0 cohort and the HEP cohort were observed (*p* = 0.53). Similarly, no correlation was shown for T-stage (according to UICC), lymph node status, histopathological tumour grade (according to WHO), tumour location, age and diabetes mellitus type 2.

**Table 1:** Expression of PPARG in the three different cohorts of CRC with different sites of distant spread (HEP and PUL) and with metastasis free survival (M0)

H-Score	M0	HEP	PUL
**Mean**	123.7	128.1	156.9
**Median**	122.5	132.5	165

**Table 2:** Clinicopathological variables and correlation with PPARG expression in the matched case control study population of 246 CRC patients. The percentages are shown in parentheses

Characteristics	Total	PPARG		*P*-Value
		low	high	
**All patients**	246 (100)	122 (49.6)	124 (50.4)	–
**Age**				
≤ 66	122 (49.6)	60 (49.2)	62 (50.0)	0.898
> 66	124 (50.4)	62 (50.8)	62 (50.0)	
**Sex**				
male	145 (58.9)	61 (50)	84 (67.7)	**0.005**
female	101 (41.1)	61 (50)	40 (32.3)	
**Tumour location**				
Colon	108 (43.9)	49 (40.2)	59 (47.6)	0.275
Rectosigmoid	22 (8.9)	14 (11.5)	8 (6.5)	
Rectum	116 (47.2)	59 (48.4)	57 (46.0)	
**pT-Stage**				
T0	2 (0.8)	0 (0)	2 (1.6)	
T1	11 (4.5)	6 (4.9)	5 (4.1)	
T2	30 (12.2)	11 (9.0)	19 (15.3)	0.343
T3	175 (71.1)	91 (74.6)	84 (67.7)	
T4	28 (11.4)	14 (11.5)	14 (11.3)	
**Tumour grade**				
Low grade	166 (67.8)	83 (68.0)	83 (67.5)	0.926
High grade	79 (32.2)	39 (32.0)	40 (32.5)	
**N-Stage**				
N negative	115 (46.7)	60 (49.2)	55 (44.4)	0.441
N positive	125 (50.8)	59 (48.4)	66 (53.2)	
Unknown	6 (2.4)	3 (2.5)	3 (2.4)	
**M-status**				
M0	82 (33.3)	47 (38.5)	35 (28.2)	Global: 0.053
HEP	82 (33.3)	43 (35.2)	39 (31.5)	M0 vs. HEP: 0.53
PUL	82 (33.3)	32 (26.2)	50 (40.3)	M0 vs. PUL: **0.019**
**Diabetes Mellitus II**				
No	191 (77.6)	96 (78.7)	95 (76.6)	0.249
Yes	33 (13.4)	13 (10,7)	20 (16.1)	
Unknown	22 (8.9)	13 (10.7)	9 (7.3)	

### Correlation of PPARG expression with overall survival using Kaplan-Meier-estimates

**Fig. 2:**
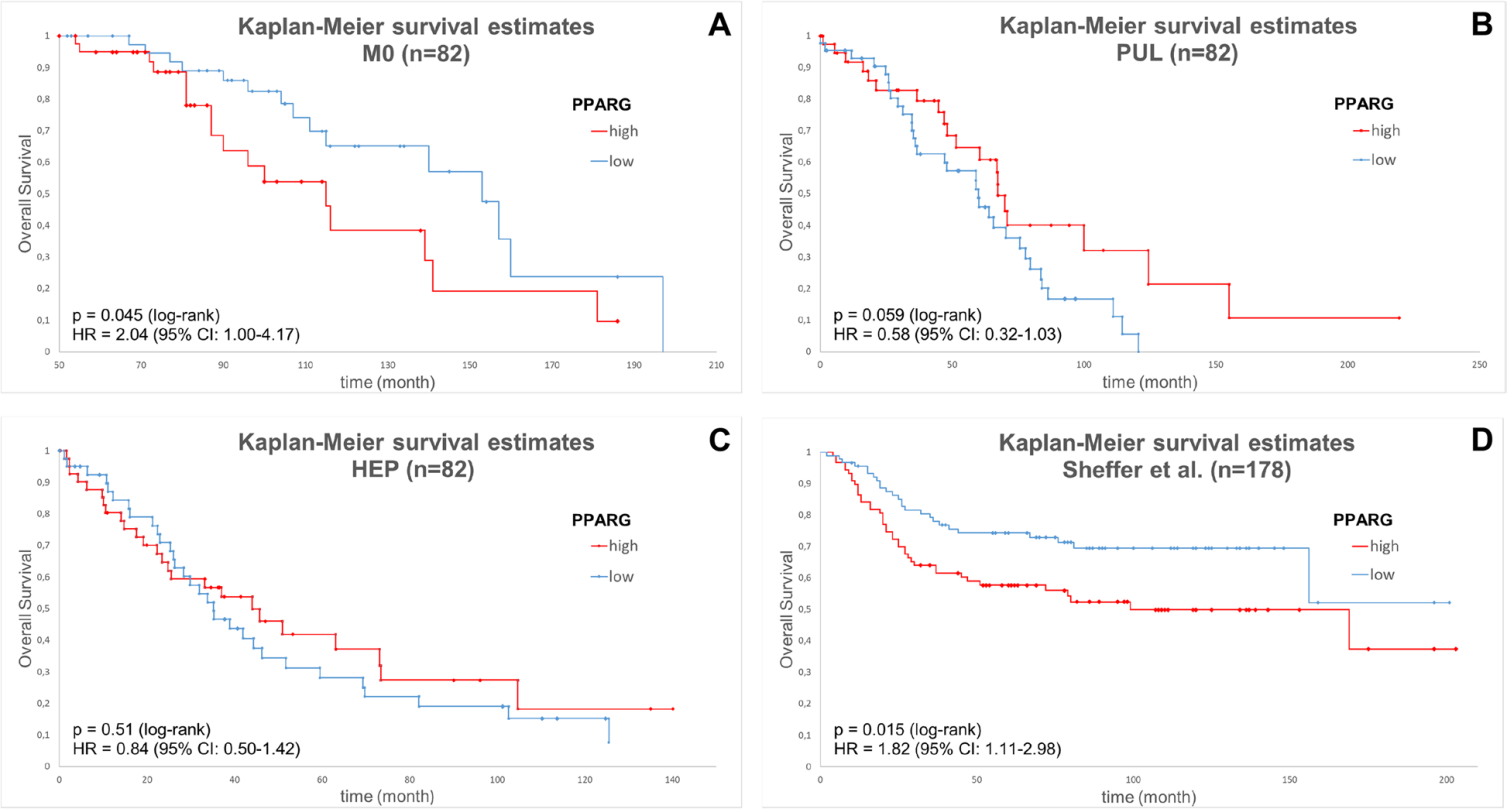
Kaplan-Meier survival estimates. Overall survival in patients with non-metastatic CRC (**A**), in patients with pulmonary metastases (**B**), in patients with hepatic metastases (**C**), in the Sheffer et al. cohort (**D**) (for statistical testing the log-rank test was used; crossed lines indicate censored cases)

To avoid bias due to different disease stages of the patients, each cohort (M0, HEP, PUL) was examined separately for the following survival analyses. The median of the corresponding cohort was set as cut-off for the classification into low or high expression of PPARG. High PPARG expression was significantly associated with poorer overall survival in the M0- subcohort (153 [133.19, 172.81] months for low expression vs 115 [92.8, 137.2] months for high expression, HR = 2.04, 95%CI 1.00–4.17, *p* = 0.045, Fig. 2 A). In contrast, in the PUL-cohort, patients with high PPARG expression showed a statistically non-significant trend towards a better overall survival compared to patients with low PPARG expression status (59.97 [43.63, 76.3] months for low expression vs 67.36 [63.07, 71.66] months for high expression, HR = 0.58, 95%CI 0.32–1.03, *p* = 0.059, Fig. 2 B). In the HEP cohort, PPARG expression did not correlate with overall survival (35.24 [24.62, 45.87] months for low expression vs 44.09 [24.51, 63.37] months for high expression, HR = 0.84, 95%CI 0.50–1.42, *p* = 0.51, Fig. 2 C). To validate the results obtained by immunohistochemistry in our own cohorts, we performed survival analyses on a publicly accessible RNAseq-based gene expression data set (*n* = 178) [23]. Low PPARG expression was significantly associated with better overall survival (HR = 1.82, 95%CI 1.11–2.98, *p* = 0.015, Fig. 2 D). Similar to our study population, the classification into low and high expression of PPARG was based on the median expression level.

### Effects of PPARG expression in colorectal cancer in vitro

#### Gene suppression of PPARG in CRC cell lines and PPARG agonist testing

**Fig. 3:**
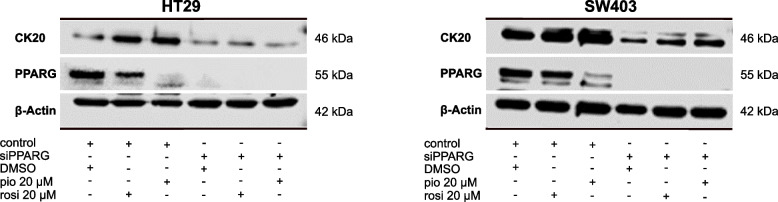
Expression level of PPARG downstream target CK20 in colorectal cancer cell lines. Colorectal cancer cell lines HT29 and SW403 were treated with the PPARG agonists pioglitazone, rosiglitazone and siPPARG. After incubation for 48 hours, the protein expression level of the PPARG downstream target CK20 was measured using immunoblotting

To evaluate the effect of PPARG on cell viability, a transient gene knockdown was established using siRNA-mediated gene silencing in colorectal cancer cell lines. For verification, protein lysates were harvested 24 and 48 hours after transfection of the HT29 and SW403 cell lines (supplemental Fig. 2). In the cell viability assays the final concentration of the siPool was kept as low as possible at 2 nM. To demonstrate that the PPARG agonists, rosiglitazone and pioglitazone act through activation of gene expression, we screened the expression of the well-known PPARG target gene of CK20 [24], 48 hours after addition of the treatment in HT29 and SW403 (Fig. 3). Pioglitazone and rosiglitazone increased the expression of CK20. Conversely, the expression of CK20 was not affected by the administration of agonists after PPARG gene knockdown. In line with these findings, the expression level of CK20 was reduced in the knockdown cell lines compared to the control cell lines.

#### Proliferation of colorectal cancer cell lines depends on PPARG activity

**Fig. 4:**
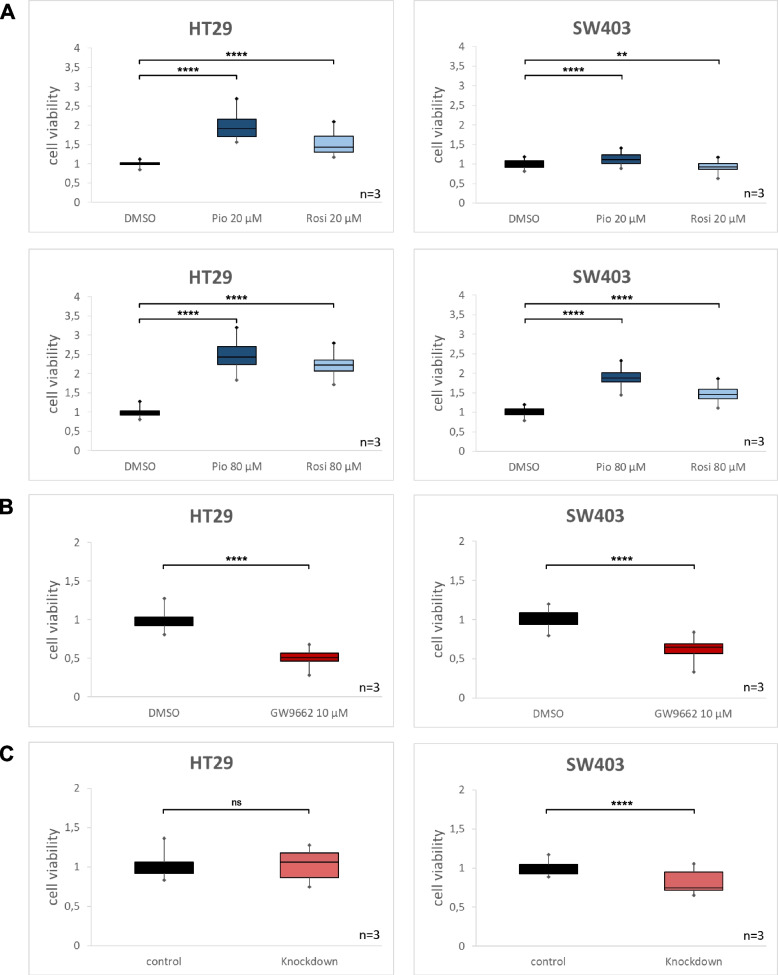
Cell viability assays. Activation of PPARG promotes proliferation in CRC cell lines (**A**). Inhibition of PPARG reduces viability in CRC cell lines (**B**). siRNA-mediated gene suppression of PPARG reduces cell viability in SW403 (**C**) (Data is presented as average ± standard deviation; statistical significance is indicated as **** *p* < 0.0001, *** *p* < 0.001, ** *p* < 0.01, * *p* < 0.05, ns non-significant; for statistical testing the t-test and Wilcoxon-Rank test were performed, both showing equal significance levels for all comparisons)

To test the effect of PPARG activity on CRC cell line proliferation, we employed the PPARG agonists pioglitazone and rosiglitazone. As expected, activation of PPARG led to a significant dose-dependent increase in proliferation of CRC cell lines (Fig. 4, A). In HT29 cells, proliferation increased 1.9-fold for pioglitazone (20 μM, *p* < 0.0001) and 1.5-fold for rosiglitazone (20 μM, *p* < 0.0001). Pioglitazone at 80 μM enhanced the proliferation 2.4-fold (*p* < 0.0001) and rosiglitazone at 80 μM 2.2-fold (*p* < 0.0001). We observed similar effects for the cell line SW403. The administration of pioglitazone 20 μM significantly increased proliferation rate by 12% compared to the DMSO group (*p* < 0.0001). Rosiglitazone 20 μM, however, reduced proliferation by 7% (*p* < 0.01). At higher concentrations, both pioglitazone 80 μM (*p* < 0.0001) and rosiglitazone 80 μM (*p* < 0.0001) increased cell proliferation by 1.9-fold and 1.5-fold, respectively. To test whether PPARG inhibition had a contrary effect on CRC cell line proliferation we employed the PPARG inhibitor GW9662. Conversely, a concentration of 10 μM caused a significant reduction in the proliferation of the CRC cell lines HT29 and SW403 (Fig. 4, B). In HT29, cell viability was about 51% compared to the control group (*p* < 0.0001). In SW403, cell viability decreased to 64% compared to untreated cells (*p* < 0.0001).

#### Knockdown of PPARG decreases the proliferation in SW403

To test whether not only the experimental stimulation or inhibition of PPARG but also its expression levels affect CRC cell line proliferation, we employed a transient gene knockdown using siPools. SiRNA-mediated suppression of PPARG caused a significant decrease in cell viability in the cell line SW403 (Fig. 4, C). Cell viability was 81% compared to control group without PPARG knockdown (*p* < 0.0001). Surprisingly, we observed no change for the cell line HT29 (*p* = 0.54).

#### Activation of PPARG increases chemotherapy sensitivity of 5-fluorouracil

**Fig. 5:**
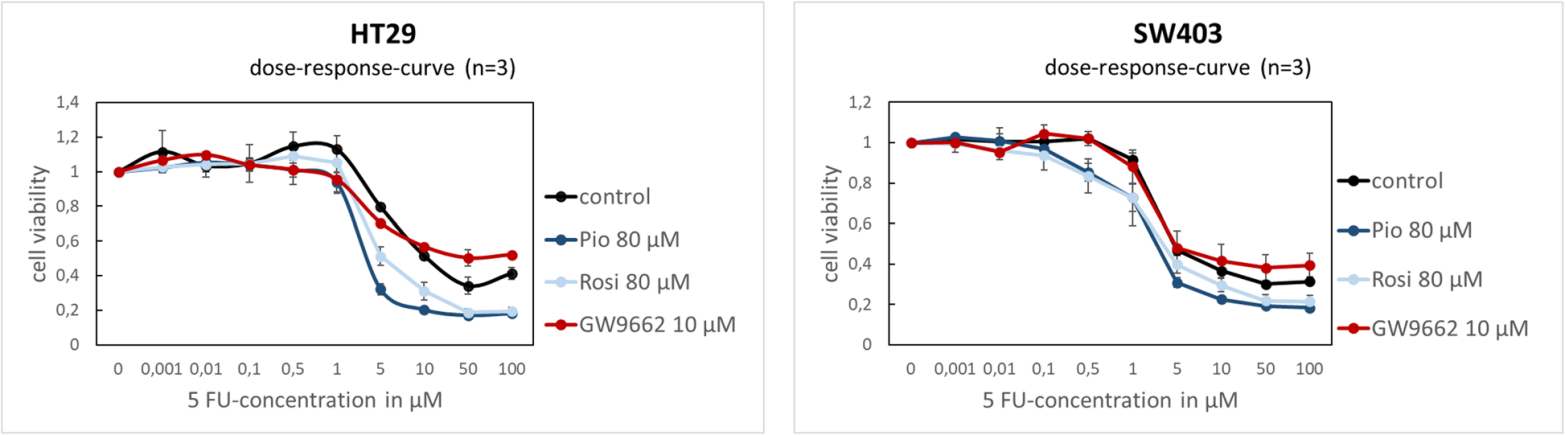
PPARG activation increases chemotherapy sensitivity of 5-Fluorouracil. Dose-response-curves for 5-Fluorouracil in cell lines HT29 and SW403 after treatment with the PPARG agonists pioglitazone and rosiglitazone, as well as the PPARG inhibitor GW9662 (statistical test results are shown in Table 3)

**Fig. 6:**
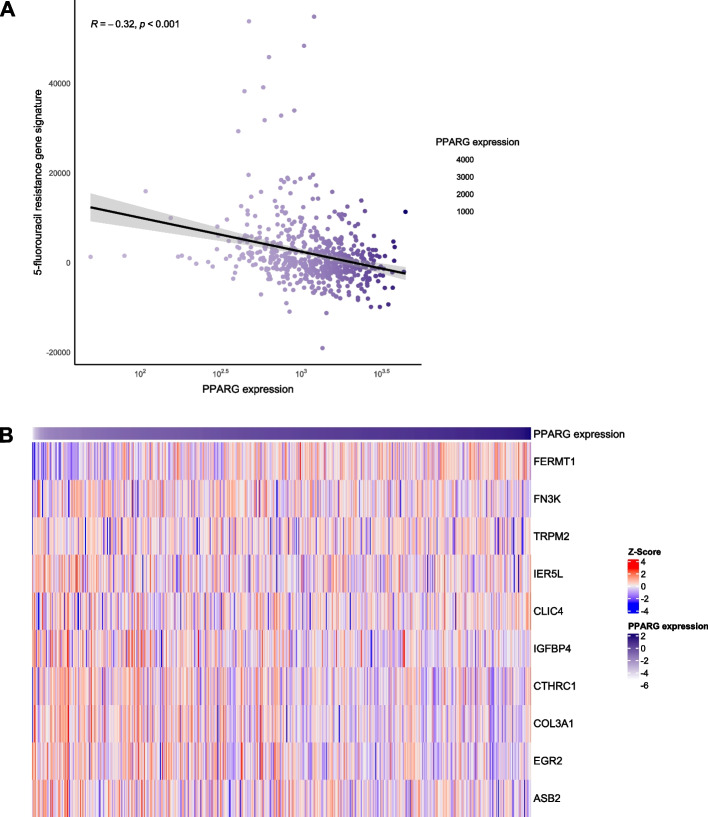
PPARG and 5-FU resistance genes. Association of PPARG expression and 10 gene-based 5-FU-resistance expression signature (**A**) and correlation of PPARG expression and the expression of 5-FU-resistance-associated genes (**B**) (for statistical testing the Pearson correlation coefficient was determined)

To test whether PPARG activity affects the response of CRC cell lines to the commonly employed chemotherapeutic drug 5-FU, we modulated PPARG activity by agonists and an inhibitor and tested the effect on CRC cell line viability when treated with 5-FU. Treatment of CRC cell lines with PPARG agonists led to a significant increase in chemotherapy sensitivity of 5-FU (Fig. 5). In untreated HT29 cells, the IC50 (half maximal inhibitory concentration) for 5-FU was approximately six times higher compared to cells pretreated with PPARG agonists (Table 3). For pioglitazone and rosiglitazone 80 μM, the IC50 was 4.4 μM and 5.3 μM, respectively. In the control group, a significant higher 5-FU concentration (31.4 μM) was necessary to achieve the identical inhibition (*p* < 0.0001). As expected, PPARG inhibitor GW9662 significantly increased the IC50 to 100.5 μM compared to untreated cells in HT29 (*p* = 0.03). Similar effects could be seen for the cell line SW403. The IC50 value was 3.8 μM for treatment with pioglitazone (*p* < 0.0001), 5.0 μM for treatment with rosiglitazone (*p* < 0.001) and 10.6 μM for untreated cells. PPARG inhibition through GW9662 led to a non-significant increase of the IC50 (18.8 μM) (*p* = 0.19) in SW403. To strengthen our in vitro findings*,* we calculated a 5-FU resistance score using expression data from the TCGA, a publicly accessible RNAseq-based gene expression data set (Fig. 6, A) [25]. Tumours with high PPARG expression showed an inverse correlation with a 5-FU resistance score and the expression levels of genes associated with 5-FU resistance (Fig. 6, B) [21].

**Table 3:** IC50 value for 5-FU after treatment with the PPARG agonists pioglitazone, rosiglitazone and the PPARG inhibitor GW9662

	Treatment	IC50 (μM) average + standard deviation	*p*-value paired t-test	*p*-value Wilcoxon-Rank test
**HT29**	Control	31.4 ± 8.2		
	Pio 80 μM	4.4 ± 0.8	**< 0.0001**	**< 0.01**
	Rosi 80 μM	5.3 ± 0.7	**< 0.0001**	**< 0.01**
	GW9662 10 μM	100.5 ± 69.9	**0.03**	**< 0.01**
**SW403**	Control	10.6 ± 1.9		
	Pio 80 μM	3.8 ± 0.6	**< 0.0001**	**< 0.01**
	Rosi 80 μM	5.0 ± 1.7	**< 0.001**	**< 0.01**
	GW9662 10 μM	18.8 ± 16.1	0.19	0.426

## Discussion

The purpose of this case control study was to investigate the effect of PPARG on CRC pathogenesis and the relevance of its expression on the clinical outcome of patients with CRC. In our case control study, we demonstrated that high expression of PPARG is significantly associated with pulmonary metastases. Metastatic status represents one of the most important factors in the outcome of patients with CRC. Patients without distant metastases have the longest overall survival, followed by patients with isolated pulmonary metastases. Patients with liver metastases have the worst prognosis among these clinical groups [26, 27]. The new findings that tumours with pulmonary metastases show stronger PPARG expressions could thus have prognostic relevance for patients with CRC. Pancione et al. [10] showed that low expression of PPARG was significantly associated with liver metastases, but patients with lung metastases were not included in their study. PPARG might have various functions depending on different stages of tumour progression, especially in relation to pulmonary metastases. Patients without distant metastases and low PPARG expression had a significantly better overall survival than patients with high PPARG expression. Moreover, our RNAseq-based expression analyses showed a highly significant better overall survival in the group of low PPARG expressing tumours, which was in line with the findings from our own cohort. Interestingly, in the analysis of the pulmonary cohort, we observed a trend reversal. Strong PPARG expression was associated with better overall survival. In contrast, Ogino et al. demonstrated that PPARG positive tumours were associated with a lower tumour-specific mortality rate. However, they did not distinguish between patients with or without distant metastases in their survival analyses [28]. In the literature, PPARG is also discussed as a tumour suppressor, especially in the early stages of colon cancer carcinogenesis [29]. In cells with non-mutated APC protein, PPARG can suppress β-catenin expression via its TCF/LEF binding domain [30]. The *APC* gene though, is mutated in more than 80% of non-hypermutated CRC [31]. Strong immunohistochemical staining of β-catenin is well known to correlate with poorer prognosis for patients with CRC, as it is a widely accepted hallmark of an activated canonical Wnt-signaling pathway [32–34]. The connection between PPARG and the Wnt-signaling pathway may be a possible explanation for the effect of PPARG on early stages of colon cancer carcinogenesis. The biological background to PPARG function in advanced tumour stages, especially metastasis, is still unclear. Also, we observed no correlation for T-stage (according to UICC), lymph node status, histopathological tumour grade (according to WHO) and tumour location. Similar results were obtained from an immunohistochemical analysis of CRC by Theocharis et al. [35].

In our in vitro experiments, we showed that activation of PPARG by pioglitazone and rosiglitazone leads to a dose-dependent increase in proliferation rate in the colorectal cancer cell lines HT29 and SW403. Likewise, Choi et al. [36] were able to show that pioglitazone promoted tumour growth in APC-mutated colorectal cells in vitro and in vivo. In mouse models, an increase in colon polyp incidence after therapy with PPARG agonists was shown [37, 38]. These results have been controversially discussed, as recent studies have mainly demonstrated an inhibitory effect on the development of aberrant crypt foci through the activation of PPARG [39, 40]. In the past, dose-dependent antiproliferative properties of PPARG agonists were shown in cell viability assays with the CRC cell lines SW480, CaCo-2 and HT29 [41, 42]. This effect could be mediated by a PPARG-dependent decrease in gene expression of the target genes COX-2 and cyclin D1 [6, 41]. However, our results from the GW9662 inhibitor cell viability assays confirmed our findings on the proliferative properties of PPARG, as inhibition of PPARG led to a decrease in the cell viability of HT29 and SW403 cells. Transient gene knockdown of PPARG also caused a decreased proliferation rate, at least in the SW403 cell line. Controversially, a germline mutation of PPARG gene is associated with tumour progression in the literature. Various loss-of-function mutations of the PPARG gene are associated with the development of CRC [14, 15]. Furthermore, in mice with hemizygous PPARG knockdown, an increased incidence of CRC was observed after inducement of colon cancer by Azoxymethane treatment [29]. On the other hand, our cell viability assays upon gene knockdown, inhibitor and agonist treatment show consistent proliferative properties of PPARG. In comparison to the literature, the effect of PPARG on proliferation remains controversial. To examine the clinical relevance of PPARG for patients with CRC, we also analysed the effect of PPARG on the chemotherapy sensitivity of 5-FU. 5-FU is a standard therapeutic agent in the multimodal therapy concept of CRC for curative as well as palliative treatment approaches [43]. We saw that activation of PPARG by pioglitazone and rosiglitazone led to a significant increase in the chemotherapy sensitivity of 5-FU in CRC cell lines. The additional administration of glitazones in patients with CRC might increase the therapeutic efficacy of 5-FU and enable a dose reduction of the cytostatic drug 5-FU. Confirmatively, further authors observed an increased 5-FU-induced apoptosis rate in CRC cells during rosiglitazone therapy [44, 45]. Based on the current literature we hypothesize that this may be mediated by increased expression of pro-apoptotic proteins such as Bax and Bad and inhibition of anti-apoptotic proteins such as Bcl-2 through PPARG activation [46]. 5-FU as a chemotherapeutic agent is well known to induce apoptosis in colorectal cells by modulating the Bcl-2 protein family [47]. This junction and the inverse correlation with the 5-FU resistance score could be an explanation for the increased sensitivity of 5-FU through PPARG activation.

## Conclusions

Taken together, high PPARG expression is associated with pulmonary metastasis. Depending on the metastatic status, PPARG seems to have a different prognostic impact on patients with CRC. Furthermore, PPARG appears to promote tumour-progressive behaviour in colorectal cancer cell lines. The increased 5-FU chemotherapy sensitivity observed under PPARG agonist administration offers a potential dual treatment approach by combining PPARG agonists and 5-FU-based chemotherapy regimens. This hypothesis should be addressed in future randomised clinical trials.

### Supplementary Information

**Additional file 1:** **S Figure 1.** PPARG expression in colorectal cancer cells

**Additional file 2:** **S Figure 2.** siRNA-mediated gene suppression of PPARG was established with siPools in cell line HT29 and SW403

**Additional file 3:** **S Table 1.** Primer sequence for rt-PCR

**Additional file 4: S Table 2.** Band density of the immunoblots (Knockdown/control (A); expression level of downstream target (B); PPARG expression in colorectal cancer cell lines (C))

**Additional file 5.**

## Data Availability

Data that support the findings of this study is available from the corresponding author upon reasonable request.
