# Supplementary figures and images for: PPARG activation promotes the proliferation of colorectal cancer cell lines and enhances the antiproliferative effect of 5-fluorouracil
Source: BMC Cancer. 2024 Feb 20;24:234. doi: 10.1186/s12885-024-11985-5 (PMC10877928; doi:10.1186/s12885-024-11985-5)

**S figure 1:** PPARG expression in colorectal cancer cells


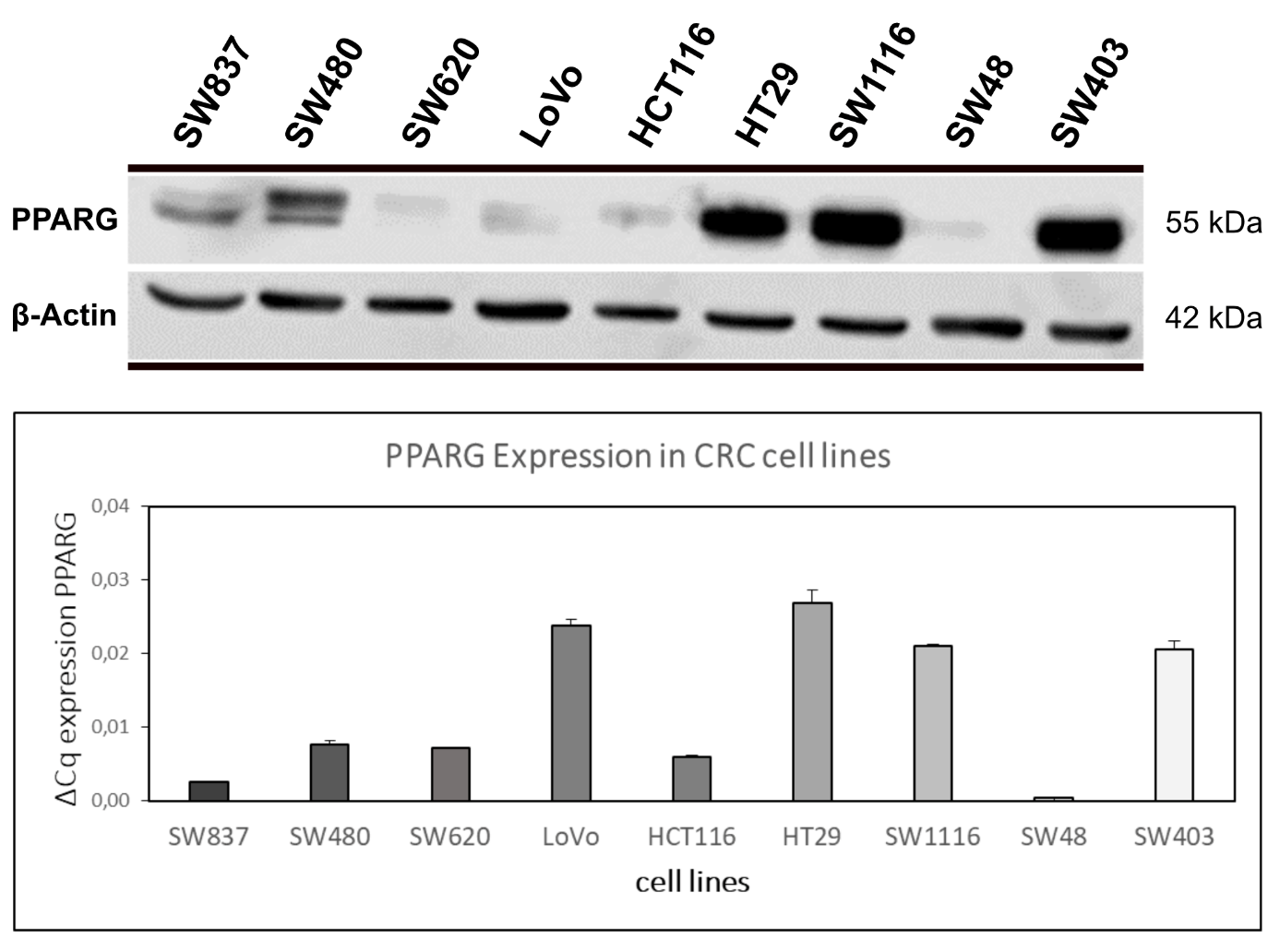

Supplement: Supplementary file 1 — Additional file 1: S Figure 1. PPARG expression in colorectal cancer cells [file 12885_2024_11985_MOESM1_ESM.docx]
