# Supplementary material for: PPARG activation promotes the proliferation of colorectal cancer cell lines and enhances the antiproliferative effect of 5-fluorouracil
Source: BMC Cancer. 2024 Feb 20;24:234. doi: 10.1186/s12885-024-11985-5 (PMC10877928; doi:10.1186/s12885-024-11985-5)

**S figure 2:** siRNA-mediated gene suppression of PPARG was established with siPools in cell line HT29 and SW403


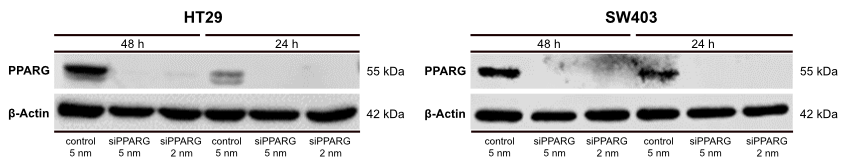

Supplement: Supplementary file 2 — Additional file 2: S Figure 2. siRNA-mediated gene suppression of PPARG was established with siPools in cell line HT29 and SW403 [file 12885_2024_11985_MOESM2_ESM.docx]
