# Supplementary material for: PPARG activation promotes the proliferation of colorectal cancer cell lines and enhances the antiproliferative effect of 5-fluorouracil
Source: BMC Cancer. 2024 Feb 20;24:234. doi: 10.1186/s12885-024-11985-5 (PMC10877928; doi:10.1186/s12885-024-11985-5)
Supplement: Supplementary file 3 — Additional file 3: S Table 1. Primer sequence for rt-PCR [file 12885_2024_11985_MOESM3_ESM.docx]

**S table 1:** Primer sequence for rt-PCR

| **GAPDH** | |
| --- | --- |
| Forward | 5‘ CTTCAACAGCGACACCCACT 3‘ (20) |
| Reverse | 5‘ CTGGTCCAGGGGTCTTACTC 3‘ (20) |
| **PPARG** | |
| Forward | 5‘ GCCCAAGTTTGAGTTTGCTG 3‘ (20) |
| Reverse | 5‘ TCAATGGGCTTCACATTCAGC 3‘ (21) |
