# Supplementary material for: PPARG activation promotes the proliferation of colorectal cancer cell lines and enhances the antiproliferative effect of 5-fluorouracil
Source: BMC Cancer. 2024 Feb 20;24:234. doi: 10.1186/s12885-024-11985-5 (PMC10877928; doi:10.1186/s12885-024-11985-5)
Supplement: Supplementary file 4 — Additional file 4: S Table 2. Band density of the immunoblots (Knockdown/control (A); expression level of downstream target (B); PPARG expression in colorectal cancer cell lines (C)) [file 12885_2024_11985_MOESM4_ESM.docx]

**S table 2:** Band density of the immunoblots (Knockdown/control (**A**); expression level of downstream target (**B**); PPARG expression in colorectal cancer cell lines (**C**))

| Part A | **HT29** | | | | | | | | | | | |
| --- | --- | --- | --- | --- | --- | --- | --- | --- | --- | --- | --- | --- |
|  | **48h** | | | | | | **24h** | | | | | |
|  | **control 5nm** | | **siPPARG 5nm** | | **siPPARG 2nm** | | **control 5nm** | | **siPPARG 5nm** | | **siPPARG 2nm** | |
|  | Area | Ratio | Area | Ratio | Area | Ratio | Area | Ratio | Area | Ratio | Area | Ratio |
| **β-Actin** | 22867 | 1 | 16724 | 1 | 17608 | 1 | 17155 | 1 | 18992 | 1 | 20898 | 1 |
| **PPARG** | 19891 | 0,870 | 170 | 0,010 | 689 | 0,039 | 6161 | 0,359 | 336 | 0,018 | 900 | 0,043 |
|  | **SW403** | | | | | | | | | | | |
|  | **48h** | | | | | | **24h** | | | | | |
|  | **control 5nm** | | **siPPARG 5nm** | | **siPPARG 2nm** | | **control 5nm** | | **siPPARG 5nm** | | **siPPARG 2nm** | |
|  | Area | Ratio | Area | Ratio | Area | Ratio | Area | Ratio | Area | Ratio | Area | Ratio |
| **β-Actin** | 24955 | 1 | 19895 | 1 | 22990 | 1 | 22258 | 1 | 22954 | 1 | 21111 | 1 |
| **PPARG** | 17372 | 0,696 | 1137 | 0,057 | 5973 | 0,260 | 21426 | 0.963 | 650 | 0,028 | 744 | 0,035 |

| Part B | **HT29** | | | | | | | | | | | |
| --- | --- | --- | --- | --- | --- | --- | --- | --- | --- | --- | --- | --- |
|  | **control** | | | | | | **siPPARG** | | | | | |
|  | **DMSO** | | **Pio 20 μM** | | **Rosi 20 μM** | | **DMSO** | | **Pio 20 μM** | | **Rosi 20 μM** | |
|  | Area | Ratio | Area | Ratio | Area | Ratio | Area | Ratio | Area | Ratio | Area | Ratio |
| **β-Actin** | 18003 | 1 | 17722 | 1 | 15018 | 1 | 18907 | 1 | 17408 | 1 | 20738 | 1 |
| **PPARG** | 29361 | 1,631 | 14875 | 0,839 | 7001 | 0,466 | 1541 | 0,082 | 136 | 0,008 | 49 | 0,002 |
| **CK20** | 7046 | 0,391 | 17746 | 1,001 | 18904 | 1,259 | 5404 | 0,286 | 6123 | 0,352 | 4025 | 0,194 |
|  | **SW403** | | | | | | | | | | | |
|  | **control** | | | | | | **siPPARG** | | | | | |
|  | **DMSO** | | **Pio 20 μM** | | **Rosi 20 μM** | | **DMSO** | | **Pio 20 μM** | | **Rosi 20 μM** | |
|  | Area | Ratio | Area | Ratio | Area | Ratio | Area | Ratio | Area | Ratio | Area | Ratio |
| **β-Actin** | 18065 | 1 | 19273 | 1 | 22243 | 1 | 15222 | 1 | 18424 | 1 | 19250 | 1 |
| **PPARG** | 17892 | 0,990 | 14439 | 0,749 | 6708 | 0,301 | 112 | 0,007 | 257 | 0,014 | 185 | 0,010 |
| **CK20** | 16355 | 0,905 | 21256 | 1,103 | 25418 | 1,143 | 5883 | 0,386 | 10794 | 0,586 | 12856 | 0,668 |

| Part C | **SW837** | | **SW480** | | **SW620** | | **LoVo** | | **HCT116** | | **HT29** | | **SW1116** | | **SW48** | | **SW403** | |
| --- | --- | --- | --- | --- | --- | --- | --- | --- | --- | --- | --- | --- | --- | --- | --- | --- | --- | --- |
|  | Area | Ratio | Area | Ratio | Area | Ratio | Area | Ratio | Area | Ratio | Area | Ratio | Area | Ratio | Area | Ratio | Area | Ratio |
| **β-Actin** | 9561 | 1 | 10902 | 1 | 11190 | 1 | 14339 | 1 | 10958 | 1 | 10620 | 1 | 10878 | 1 | 14034 | 1 | 12058 | 1 |
| **PPARG** | 10910 | 1,141 | 16552 | 1,518 | 1605 | 0,143 | 2749 | 0,192 | 2506 | 0,229 | 27562 | 2,595 | 32565 | 2,994 | 1336 | 0,095 | 28635 | 2,375 |
