# Supplementary material for: PPARG activation promotes the proliferation of colorectal cancer cell lines and enhances the antiproliferative effect of 5-fluorouracil
Source: BMC Cancer. 2024 Feb 20;24:234. doi: 10.1186/s12885-024-11985-5 (PMC10877928; doi:10.1186/s12885-024-11985-5)
Supplement: Supplementary file 5 — Additional file 5. [file 12885_2024_11985_MOESM5_ESM.docx]

Supplemental: Original Western Blot Images for S figure 1

SW620

LoVo

HCT116

HT29

SW1116

SW48

SW837

SW480

HCT116

LoVo

HT29

SW1116

SW48

SW620

SW480

SW837

SW403

SW403


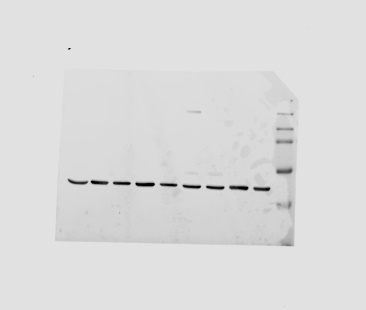

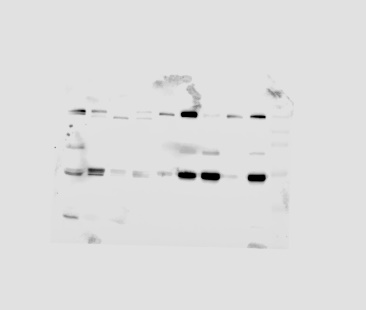


CK20 PPARG ß-Actin (CK20)

SW403

PPARG ß-Actin

Supplemental: Original Western Blot Images for S figure 2


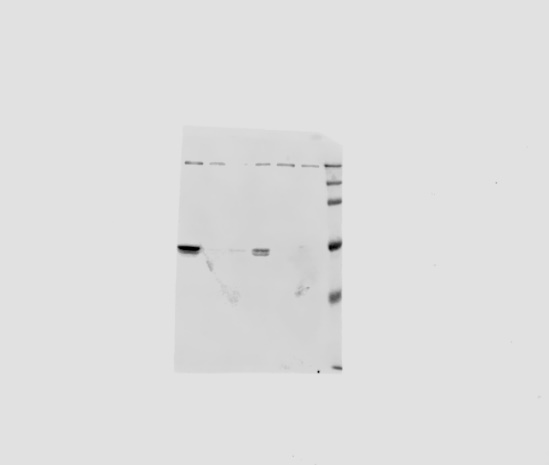
HT29


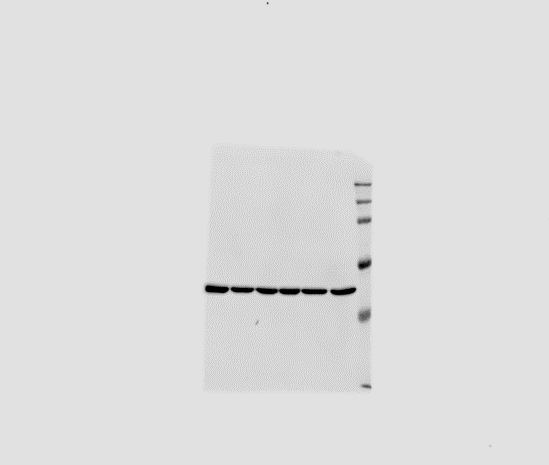


PPARG ß-Actin

SW403


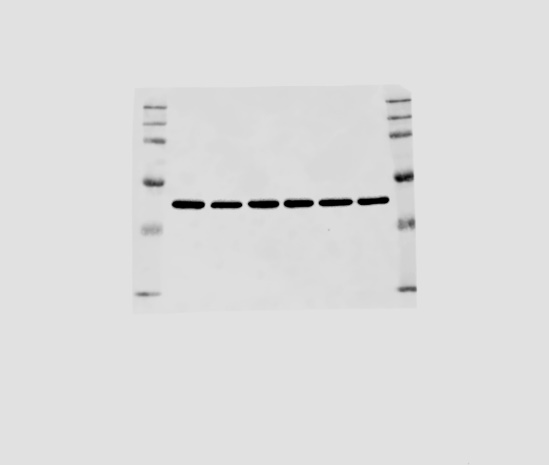

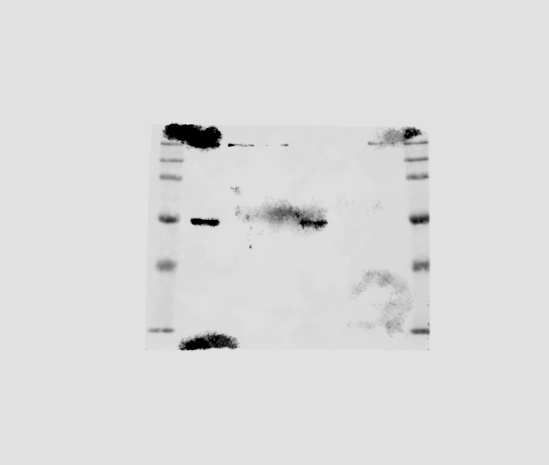


PPARG ß-Actin

Supplemental: Original Western Blot Images for figure 3


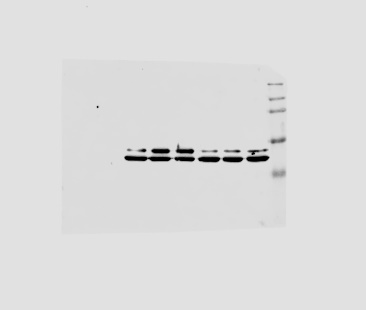

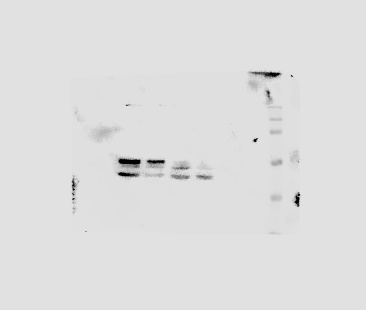

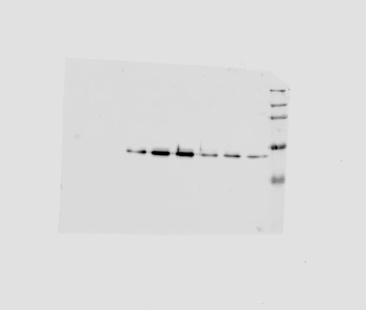
HT29

CK20 PPARG ß-Actin (CK20)


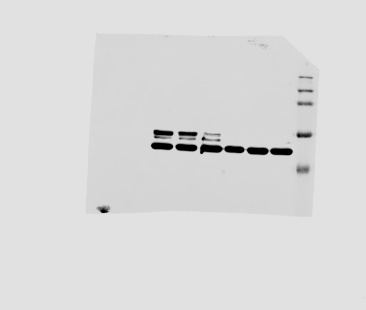

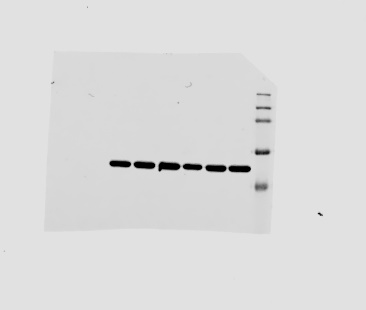

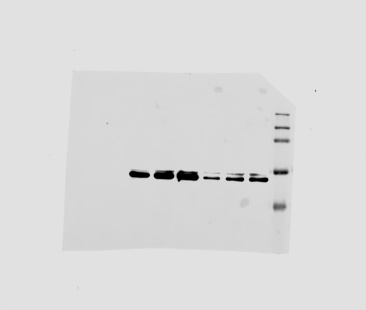
SW403

CK20 PPARG (ß-Actin) ß-Actin
